# Genetic diversity and symbiotic effectiveness of *Phaseolus vulgaris*-nodulating rhizobia in Kenya

**DOI:** 10.1016/j.syapm.2018.02.001

**Published:** 2018-07

**Authors:** George M. Mwenda, Graham W. O’Hara, Sofie E. De Meyer, John G. Howieson, Jason J. Terpolilli

**Affiliations:** Centre for Rhizobium Studies, Murdoch University, 90 South Street, Murdoch, WA 6150, Australia

**Keywords:** *Phaseolus vulgaris*, Nodulation, *Rhizobium*, Phylogeny, MLSA

## Abstract

*Phaseolus vulgaris* (common bean) was introduced to Kenya several centuries ago but the rhizobia that nodulate it in the country remain poorly characterised. To address this gap in knowledge, 178 isolates recovered from the root nodules of *P. vulgaris* cultivated in Kenya were genotyped stepwise by the analysis of genomic DNA fingerprints, PCR-RFLP and 16S rRNA, *atpD*, *recA* and *nodC* gene sequences. Results indicated that *P. vulgaris* in Kenya is nodulated by at least six *Rhizobium* genospecies, with most of the isolates belonging to *Rhizobium phaseoli* and a possibly novel *Rhizobium* species. Infrequently, isolates belonged to *Rhizobium paranaense*, *Rhizobium leucaenae*, *Rhizobium sophoriradicis* and *Rhizobium aegyptiacum*. Despite considerable core-gene heterogeneity among the isolates, only four *nodC* gene alleles were observed indicating conservation within this gene. Testing of the capacity of the isolates to fix nitrogen (N_2_) in symbiosis with *P. vulgaris* revealed wide variations in effectiveness, with ten isolates comparable to *Rhizobium tropici* CIAT 899, a commercial inoculant strain for *P. vulgaris*. In addition to unveiling effective native rhizobial strains with potential as inoculants in Kenya, this study demonstrated that Kenyan soils harbour diverse *P. vulgaris*-nodulating rhizobia, some of which formed phylogenetic clusters distinct from known lineages. The native rhizobia differed by site, suggesting that field inoculation of *P. vulgaris* may need to be locally optimised.

## Introduction

*Phaseolus vulgaris* (common bean) is a grain legume that provides dietary protein to millions of people around the world [10]. Despite its high nutritional and economic significance, the productivity of *P. vulgaris* lags behind most other crop legumes [11], with nitrogen deficiency a key limiting factor [10], [21]. Since this legume can fix nitrogen (N_2_) in symbiosis with certain soil bacteria known as rhizobia, harnessing this interaction has the potential to significantly improve the productivity of *P. vulgaris* [24].

*P. vulgaris* is nodulated by at least 27 species of rhizobia across four bacterial genera and is therefore considered a promiscuous host [13], [40]. *P. vulgaris*-nodulating rhizobia have been extensively characterised in many areas, especially in the Mesoamerican and Andean centres of common bean diversification [1], [33], [47], [56]. However, as microbial species are spatially distributed according to niche selection pressure [35], the diversity of rhizobia isolated from *P. vulgaris* centres of origin and areas of recent introductions is likely to vary considerably. Also, in regions where long periods have elapsed since *P. vulgaris* was introduced, established landraces may influence the composition of rhizobia since plant genotypes can show a preference for certain rhizobial groups [1].

Kenya is an example of a region of *P. vulgaris* introduction where the diversity and symbiotic effectiveness of the native rhizobia is poorly understood. *P. vulgaris* was introduced in the country ca. 500 years ago [22] and numerous primary and secondary landraces are currently cultivated [5]. Nodulation occurs with native rhizobia that may fix N_2_ sub-optimally [3], [29], [39], leading to occasional attempts at inoculation with *Rhizobium tropici* CIAT 899 [7]. This strain was originally isolated from *P. vulgaris* growing in Colombia and although it tolerates high temperatures, low soil pH and is genetically stable [26], [34], it fails to establish in some areas due to poor adaptability to the edaphic conditions [2], [38]. Developing an inoculant from rhizobia isolated from *P. vulgaris* growing in Kenya may provide a strain that is better adapted to the prevailing conditions.

Very few studies have genetically characterised the rhizobia that nodulate *P. vulgaris* in Kenya. In the most detailed to date, rhizobia nodulating *P. vulgaris* at two sites grouped with members of *Rhizobium etli*, *Rhizobium leguminosarum* and *R. tropici* based upon host range, *nifH* copy number and genomic DNA restriction fragment fingerprints [3]. In the more than 20 years since this study was conducted, there have been substantial changes to the taxonomy of rhizobia and new tools such as multilocus sequencing analysis now allow better discrimination between taxa [4], [45], [48].

To characterise rhizobia nodulating *P. vulgaris* in Kenya, 178 rhizobial strains isolated from *P. vulgaris* in different agro-ecologies were genotyped and a subset assessed for N_2_ fixation on the Kenyan *P. vulgaris* cultivar KK08. Strains were genetically characterised by DNA fingerprinting, restriction fragment length polymorphism (RFLP) of 16S rRNA gene (PCR-RFLP) and sequencing of 16S rRNA, *recA*, *atpD*, and *nodC* genes. The symbiotic performance of the strains was evaluated through the comparison of dry shoot weights of plants grown in controlled glasshouse conditions.

## Materials and methods

### Sampling sites and isolation of rhizobia

Root nodules were sampled from cultivated *P. vulgaris* at 16 sites in five Kenyan counties (Nairobi, Kiambu, Meru, Siaya and Busia) in 2011 and 2012 (Table S1 and Fig. S1). The sites had no known history of rhizobial inoculation and represent some of the agro-ecologies in which *P. vulgaris* is cultivated in Kenya. At each location, at least three root nodules were sampled from as many as five plants and nodules pooled in airtight vials containing silica gel [23]. Bacteria were isolated as previously described [25], authenticated on *P. vulgaris* and pure cultures stored at −80 °C in Tryptone Yeast (TY) broth containing 15% (v/v) glycerol [25].

### Preliminary analysis of diversity by DNA fingerprinting and RFLP-PCR of 16S rRNA gene

Strains were cultured on TY agar plates for three days at 28 °C and DNA extracted from isolated bacterial colonies by alkaline lysis [6] prior to storage at −20 °C. To assess strain diversity and identify closely related isolates or clones, DNA fingerprinting was performed using a *nif*-directed RP01 primer [49] as per conditions indicated in Table S2. The PCR products were then separated on 2% (w/v) agarose gels and images captured [46]. The similarities among digitised profiles were calculated using the Pearson correlation in Bionumerics v5.1 (Applied Maths, Belgium) and an average linkage (UPGMA) dendrogram derived from the patterns. Based on the reported relatedness of banding patterns of clonal strains with common DNA fingerprinting techniques [12], [16], [19], strains showing >80% similarity in their banding patterns were assigned the same ‘RP01-PCR group’.

Strain diversity was further explored by RFLP analysis of PCR-amplified 16S rRNA gene. The 16S rRNA gene of strains selected from different RP01-PCR groups was amplified using the 27F and 1492R primer set [32] (Table S2). Purified PCR products were then digested separately with HaeІІІ, MspІ, HhaІ and HinfІ (Promega Corporation), separated on agarose gels and the resulting banding patterns scored to assign strains to 16S RFLP groups [40].

### Amplification and sequencing of 16S rRNA, *recA*, *atpD* and *nodC* genes

From the 16S RFLP groups obtained, representative strains were selected for amplification and sequencing of partial 16S rRNA, *recA*, *atpD* and *nodC* genes, with primers and protocols given in Table S2. Following Sanger sequencing (Australian Genome Research Facility, Perth, Australia), sequences were edited and assembled with Geneious software (Biomatters Ltd, NZ) and deposited into GenBank (Table S1).

The 16S rRNA sequences were used to search for bacterial type strains with highly similar 16S rRNA genes in EzTaxon-e [31] and sequences of type strains showing ≥98.65% similarity [30] retrieved for use in phylogenetic analyses (accession numbers are listed in Table S1). For the remaining genes, reference sequences were obtained from GenBank by searching for corresponding sequences of related type strains, as determined by 16S rRNA. Sequences were aligned using MEGA6 and phylogenetic trees constructed using the Maximum Likelihood method with best fit models selected based on AICc values (Akaike Information Criterion, corrected), Maximum Likelihood values (lnL), and the number of parameters (including branch lengths) [53]. Bootstrap analysis with 1000 replicates was performed to assess the support of the clusters. The *recA* and *atpD* genes produced congruent trees (Figs. S2 and S3) and were therefore concatenated using Geneious software. The final alignment (736 bp) was analysed as described above.

### Assessment of N_2_ fixation

All isolates analysed by MLSA were evaluated for symbiotic N_2_ fixation with *R. tropici* CIAT 899 [34] and *R. leguminosarum* sv. phaseoli 8002 [28] as reference strains on *P. vulgaris* cultivar KK08. Growth experiments were conducted in a glasshouse under natural light. Plants were grown in 3.5 L free-draining pots with steam-sterilized fine vermiculite, where growth of the legume is limited by N-deficiency except when nodulated by a rhizobial strain capable of fixing N_2_ [59]. Rhizobia cultured in TY broth were washed and suspended in sterile water and then a 1 mL aliquot (approximately 5 × 10^8^ cells) applied to each surface-sterilised pre-germinated seed, while un-inoculated N-free treatments received 1 mL of sterile water [40]. All treatments were replicated in three pots, each pot containing three plants that were thinned to two plants seven days after inoculation. Each pot was supplied with 150 mL of sterile N-free nutrient solution [9] per week and with sterile water as required. Plants were harvested 42 days after inoculation, shoots excised and dried for 48 h at 60 °C, then weighed.

Shoot dry weights (SDW) of inoculated plants were expressed as a percentage of the mean weight of the CIAT 899 treatment and categorized as effective (≥75% of CIAT 899 SDW), partially effective (74%–50%), poorly effective (49–25%), or ineffective (≤24%) as described previously [54]. Analysis of variance (ANOVA) was performed using SPSS version 22 (IBM Corp, released 2013) and Fisher’s LSD calculated when ANOVA was found to be significant (p < 0.05). Isolates were also grouped by taxa and by differences in their *nodC* sequences and N_2_ fixation analysed by ANOVA.

## Results

### Preliminary analysis of diversity

A total of 178 isolates recovered from *P. vulgaris* nodules were authenticated as rhizobia. These formed 87 RP01-PCR groups (Table S1), with the high number of groups suggesting the isolates were genetically diverse. To further explore this diversity, representative isolates were selected from these groups and analysed by PCR-RFLP. Based on shared banding patterns for four restriction enzymes, 56 isolates grouped into PCR-RFLP Group 1; 27 into Group 2; four into Group 3; and one into Group 4 (Table S1). From the four PCR-RFLP groups, 36 isolates were then selected for detailed phylogenetic analysis.

### Phylogeny based on 16S rRNA gene

**Fig. 1:**
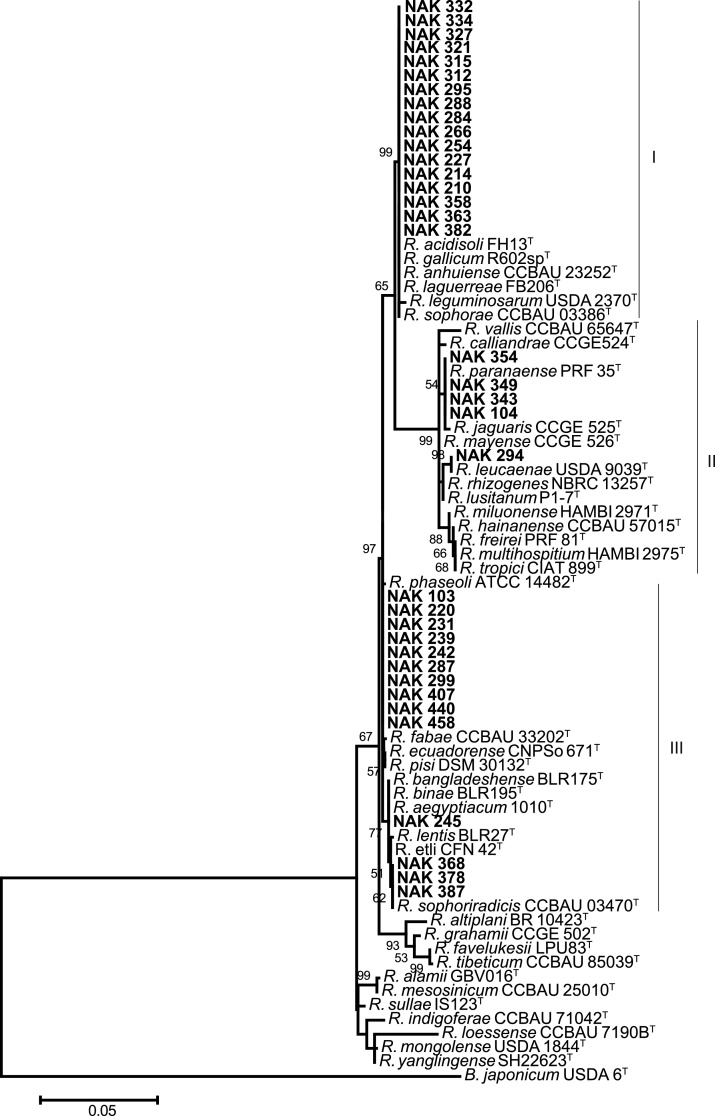
Phylogenetic tree of the 16S rRNA gene from 36 isolates (in bold) and type strains of closely related species constructed using the Maximum Likelihood method based on the Tamura 3-parameter model in MEGA6 [53]. There was a total of 1305 positions in the final dataset, and node supports higher than 50% are labelled with a bootstrap value (1000 replicates). The sequence of *Bradyrhizobium japonicum* USDA 6^T^ was included as an outgroup. Bar indicates five nucleotide substitutions per 100 nucleotides.

The analysis of 16S rRNA gene sequences separated the 36 isolates into three clades (Fig. 1). Seventeen isolates were in Clade I, 14 in Clade II, and five in Clade III (Fig. 1). Since genus affiliation is reliably established using 16S rRNA gene sequences [18], [55], phylogenetic analysis of this gene revealed that all the isolates belonged to the genus *Rhizobium*. However, due to the conserved nature of the 16S rRNA gene within this group [27], sequencing of housekeeping genes was carried out to further resolve the taxonomy of the isolates.

### Phylogeny based on *recA* and *atpD* genes

**Fig. 2:**
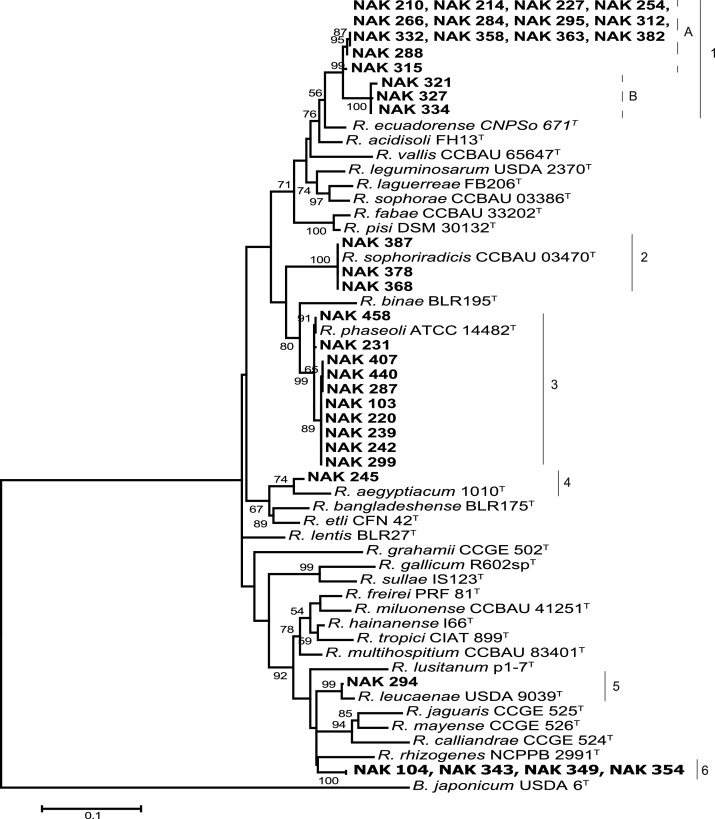
The phylogenetic relationship between the study isolates (in bold) and type strains of closely related species based on concatenated *recA* and *atpD* genes. The evolutionary history was inferred using the Maximum Likelihood method based on the General Time Reversible model in MEGA6 [53]. There was a total of 736 positions in the final dataset, and node supports higher than 50% are labelled with a bootstrap value (1000 replicates). The sequence of *B. japonicum* USDA 6^T^ was included as an outgroup. Bar indicates 10 nucleotide substitutions per 100 nucleotides.

The analysis of concatenated sequences of housekeeping genes *recA* and *atpD* provided further discrimination of the isolates, resulting in six separate clades (Fig. 2). The 17 isolates in Clade I based on the 16S rRNA gene, formed a group containing two well-supported (99% bootstrap value) sub-clades, 1A and 1B (Fig. 2). Isolates in these sub-clades were closest to CNPSO 671^T^, the type strain of *Rhizobium ecuadorense* reported to nodulate *P. vulgaris* in Ecuador and Mexico [47]. Maximum nucleotide similarities between sub-clade 1A isolates (14 isolates) and *R. ecuadorense* CNPSO 671^T^ ranged between 95.9% and 96.2% while similarities between sub-clade 1B isolates (3 isolates) and CNPSO 671^T^ were ≤94%. Within the *Rhizobium* genus, concatenated *recA*-*atpD* genes differed by as little as 2.1% (e.g. between *Rhizobium fabae* CCBAU 33202^T^ and *Rhizobium pisi* DSM 30132^T^) and 14 of the 28 *Rhizobium* spp. type strains analysed showed *recA*-*atpD* sequence divergence of <6% with any type strain. Therefore, the sequence divergence of 3.8 to >6% observed between sub-clade 1A and 1B isolates and current type strains suggests these isolates may belong to a new species. Isolates in this group were found in 10 of the 16 study sites, making it the most prevalent taxonomic group.

Three isolates (NAK 368, NAK 378 and NAK 387) (Clade 2, Fig. 2) had a 100% *recA*-*atpD* nucleotide identity to *Rhizobium sophoriradicis* CCBAU 03470^T^, isolated from the root nodule of the medicinal legume *Sophora flavescens* in China [27] and were consequently identified as *R. sophoriradicis*. The three isolates were recovered from a single site. Another ten isolates had high sequence similarity (99%–100%) to *Rhizobium phaseoli* ATCC 14482^T^ (Clade 3) and therefore belonged to *R. phaseoli*. *R. phaseoli* was recovered from seven sites. NAK 245, had a 96% nucleotide identity to *Rhizobium aegyptiacum* 1010^T^, isolated from *Trifolium alexandrinum* in Egypt [52] (Clade 4; Fig. 2), however the relatively high sequence divergence between the two strains means that NAK 245 was only tentatively identified as *R. aegyptiacum*.

NAK 294 was 98.7% similar to *Rhizobium leucaenae* USDA 9039^T^ (Clade 5) and was identified as belonging to *R. leucaenae,* a broad-host-range species that nodulates *P. vulgaris* and several tropical leguminous trees [48]. Four isolates (NAK 104, NAK 343, NAK 349 and NAK 354) formed a monophyletic group affiliated to the type strains of several species related to *R. tropici* (Clade 6). This monophyletic group had a *recA* sequence 97.2% identical to that of PRF 35^T^, the type strain of **Rhizobium* paranaense* that nodulates *P. vulgaris* in Brazil [15]. The *atpD* sequence of PRF 35^T^ was not available, and the strain was therefore not included in the analysis of the concatenated *recA*-*atpD* genes. However, the observed high *recA* and 16S rRNA gene similarities between PRF 35^T^ and the four isolates suggests that the isolates likely belong to *R. paranaense*. Isolates in this group were recovered from three sites, making it the third most prevalent group.

Overall, at least six rhizobial species were found to nodulate *P. vulgaris* in Kenya. Ten isolates were definitively assigned to *R. phaseoli*, three isolates to *R. sophoriradicis* and one to *R. leucaenae*, while four isolates were tentatively assigned to *R. paranaense* and one to *R. aegyptiacum*. Lastly, seventeen isolates had low similarity in their *recA* and *atpD* gene sequences to current type strains and may constitute a novel species.

### Symbiotic diversity

**Fig. 3:**
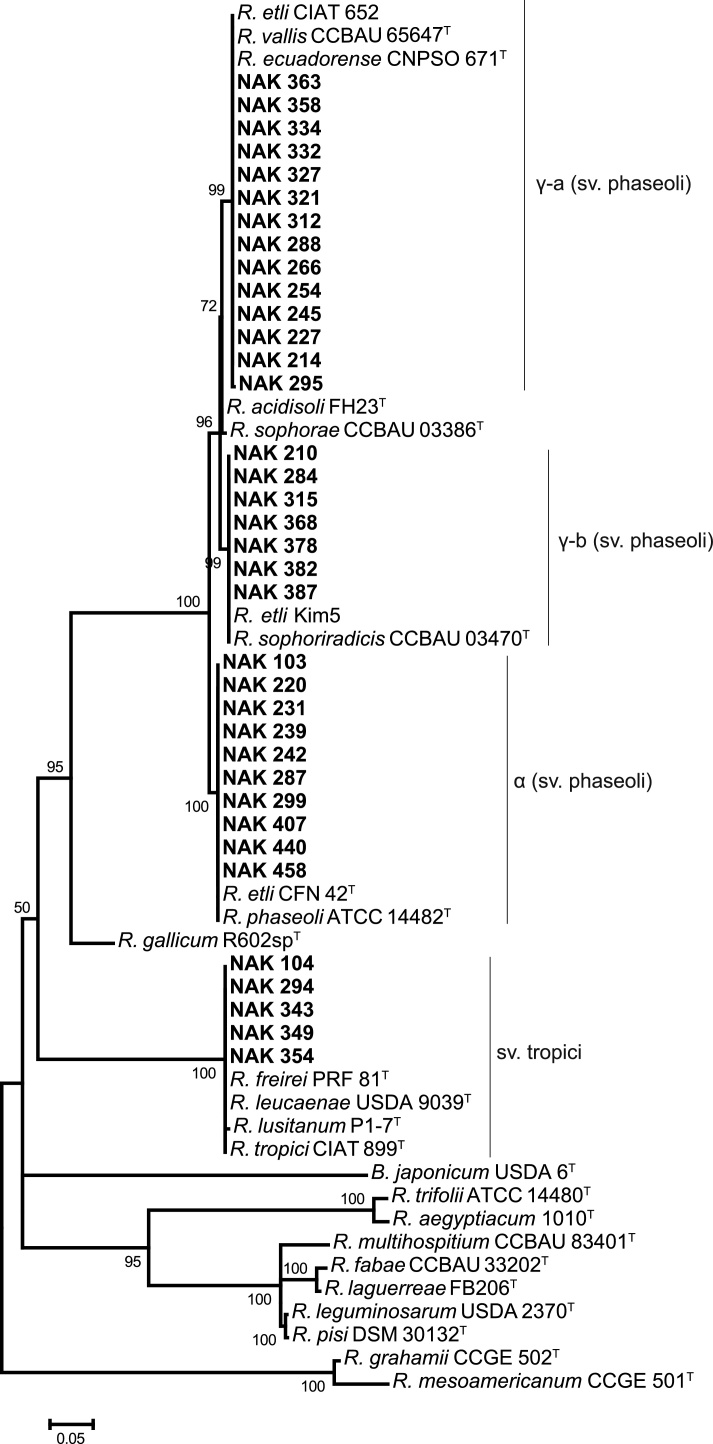
Phylogenetic tree of the *nodC* gene from 36 isolates (in bold) and reference strains constructed using the Maximum Likelihood method based on the Tamura 3-parameter model in MEGA6 [53]. There was a total of 504 positions in the final dataset, and node supports higher than 50% are labelled with a bootstrap value (1000 replicates). The sequence of *B. japonicum* USDA 6^T^ was included as an outgroup. Bar indicates five nucleotide substitutions per 100 nucleotides.

The symbiotic diversity among the isolates was assessed through the sequencing and analysis of the *nodC* gene, which is a common way of assigning rhizobia to symbiovars [17], [50], [51]. Based on partial *nodC* sequences, the 36 isolates grouped into two clades that correspond to symbiovars phaseoli and tropici (Fig. 3. The phaseoli clade had three sub-clades, designated γ-a, γ-b and α (adapted after [1], [51]), that represent different alleles of the *nodC* gene. These alleles have previously been reported to be associated with rhizobial preference for geographically cognate *P. vulgaris* [1]. Fourteen isolates in the γ-a sub-clade showed 100% sequence similarity in the *nodC* gene sequence with *Rhizobium vallis* CCBAU 65647^T^, *R. ecuadorense* CNPSO 671^T^ and *R. etli* (phaseoli) CIAT 652, which are *P. vulgaris*-nodulating strains isolated from China, Ecuador, and Costa Rica, respectively [20], [47], [57]. This allele (γ-a) was the most prevalent and was identified in isolates belonging to the putative novel species and to *R. aegyptiacum*.

A further seven isolates, in γ-b, shared 100% sequence similarity to the *nodC* of the *P. vulgaris*-nodulating *R. sophoriradicis* CCBAU 03470^T^ isolated from the medicinal legume *Sophora flavescens* in China [27] and *R. etli* KIM5, a well-characterized *P. vulgaris*-nodulating strain from the USA [20]. Isolates carrying *nodC* type γ-b belonged to the putative novel species or to *R. sophoriradicis*. The last of the phaseoli subgroups, α, comprised ten isolates with *nodC* sequences identical to those of the well-characterised, *P. vulgaris*-nodulating *R. etli* CFN 42^T^ from Mexico [43] and *R. phaseoli* ATCC 14482^T^. Allele α was the second most prevalent allele and was identified in isolates belonging to *R. phaseoli.*

Finally, five isolates had *nodC* sequences 100% identical to those of *R. tropici* CIAT 899^T^ and *R. leucaenae* USDA 9039^T^, forming a clade that corresponds to symbiovar tropici (Fig. 3). Strains in this symbiovar are often broad host strains that nodulate *P. vulgaris* and several tropical leguminous trees [41].

Except for *nodC* α (identified in *R. phaseoli*), all other alleles were identified in multiple taxonomic groups, although only the putative novel species had isolates with different *nodC* alleles (γ-a and γ-b). Overall, a limited symbiotic diversity was observed in the relatively chromosomally more diverse rhizobia that nodulate *P. vulgaris* in Kenya.

### N_2_ fixation

**Fig. 4:**
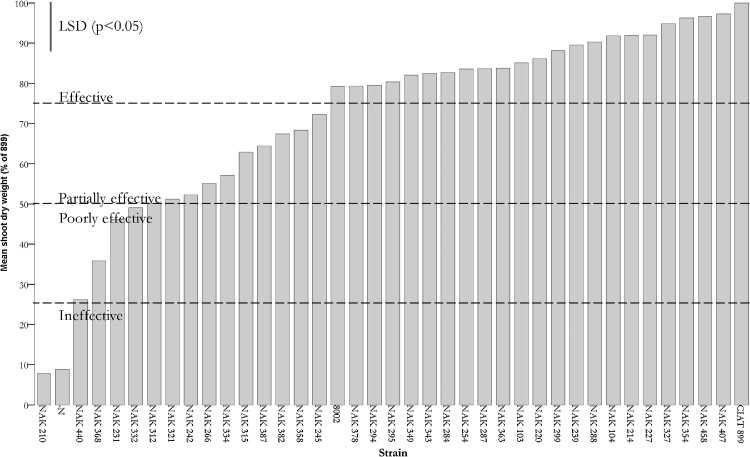
Mean shoot dry weights of *P. vulgaris* cv. KK08 inoculated with 36 rhizobial isolates from Kenya expressed as a percentage of CIAT 899 treatment. N denotes the un-inoculated treatment. All plants were maintained with nitrogen-free growth media. Data are means of six plants, harvested 42 days after inoculation.

Analysis of shoot dry weight production of *P. vulgaris* inoculated with the 36 rhizobial isolates revealed a wide range of variation in symbiotic N_2_ fixation, with a 12.5-fold difference in shoot dry weights between the most and least effective (Fig. 4). One isolate was ineffective, four poorly effective, ten partially effective and 21 were effective (Fig. 4). Ten of the 21 effective isolates induced biomass comparable to that of the current commercial inoculant strain for *P. vulgaris* in Kenya, *R. tropici* CIAT 899 (LSD, p > 0.05). These ten isolates were NAK 407, NAK 458, NAK 354, NAK 327, NAK 227, NAK 214, NAK 104, NAK 288, NAK 239 and NAK 299 and have potential for development into Kenyan *P. vulgaris* inoculants.

The analysis of effectiveness by taxonomic groups revealed significant differences based on taxonomic grouping (*F*_3,201_ = 4.100, *p* *=* 0.007). *R. paranaense* isolates were the most effective ($\bar{x}$ = 89 ± 3.6 (SEM)), followed by *R. phaseoli* ($\bar{x}$ = 81 ± 3), while those of the novel lineage ($\bar{x}$ = 73 ± 2) and *R. sophoriradicis* ($\bar{x}$ = 69 ± 5) were the least effective. Only single isolates belonged to *R. aegyptiacum* and *R. leucaenae* and these groups were therefore not included in the analysis. N_2_ fixation also differed by the *nodC* type (*F*_3,212_ = 8.901, *p <* 0.001). Isolates with *nodC* type γ-b resulted in N_2_ fixation ($\bar{x}$ = 61.7 ± 4.5) which was significantly lower than that of isolates with *nodC* types tropici, α and γ-a ($\bar{x}$ of 86 ± 3.0, 81 ± 3 and 77 ± 2 respectively). Isolates belonging to the novel group had either *nodC* type γ-b or γ-a with those carrying type γ-b being of lower effectiveness (57 ± 7 vs 78 ± 2). The other poorly effective taxonomic group (*R. sophoriradicis*) had all isolates carrying *nodC* type γ-b. These results suggest that *nodC* type γ-b may be associated with poor N_2_ fixation in *P. vulgaris* cv. KK08.

## Discussion

Using a four-tiered reductionist approach, genetically diverse rhizobia were found to nodulate and fix N_2_ with *P. vulgaris* in Kenyan soils. An initial analysis of genetic relatedness, sifted 178 isolates into 36 that were further characterized by 16S rRNA, *recA*, *atpD* and *nodC* gene sequence analysis. The isolates grouped into at least six rhizobial species that include *R. phaseoli*, *R. sophoriradicis, R. leucaenae*, *R. aegyptiacum*, *R. paranaense* and possibly a new species.

Most of the isolates belonged to *R. phaseoli* or to the putative novel lineage, which contrasts with findings in the centres of *P. vulgaris* domestication, where *R. etli* is the main microsymbiont [1], [33]. Although diverse *P. vulgaris*-nodulating rhizobia that differ from those in the centres of origin are known to occur across the globe [33], this was the first detailed account of *P. vulgaris*-nodulating rhizobia in Kenya. The diverse and distinct lineages observed in Kenya, an area of *P. vulgaris* introduction, are consistent with the distance-decay theory of biological similarity in which genetic diversity increases with increasing geographic distance from a source, depending on dispersal limitation and niche difference [8], [36].

Interestingly, despite the observed divergence in the core genomes, isolates harboured highly conserved *nodC* genes, with only four alleles identified. These same alleles are present in numerous *P. vulgaris*-nodulating species [4], [27], [50], [51], suggesting that the *P. vulgaris*-nodulating strains have arisen from chromosomal speciation accompanied by the inheritance of conserved symbiotic genes acquired through horizontal gene transfer. Indeed, recent genomic analysis of sympatric populations of *P. vulgaris*-nodulating rhizobia from Mexico indicates that while chromosomal and accessory plasmid diversity varies greatly among isolates, symbiotic plasmids tend to be conserved across multiple *Rhizobium* spp. [42]. This mode of evolution fits the recurrent niche invasion model of speciation, in which a lineage diversifies over time, but with repeated loss and acquisition of conserved niche-determining genes [58].

The distribution of rhizobial taxonomic groups was found to vary among the sampling sites, with eight of the 16 sites having only one group. As the sampling sites were heterogeneous for rain, soil characteristics and elevation (Table S1, Fig. S1), all important factors in the survival of rhizobia [26], [60], this distribution of the taxonomic groups likely reflects their adaption to the physical environments. This finding on spatial distribution of taxonomic groups amongst the sampling sites indicates a need for geographically-targeted inoculation strategies, since rhizobial groups differ in competitiveness for nodulation, a characteristic that affects the establishment of inoculant strains.

Ten isolates fixed as much N_2_ as the commonly used commercial inoculant for *P. vulgaris*, *R. tropici* CIAT 899, and these are now candidate inoculant strains. The candidate strains were isolated from diverse agro-ecological zones differing in soil types, rainfall, and temperatures (Table S1, Fig. S1) and consequently may display adaptability to specific environmental stresses present in those areas. These strains could be developed into inoculants for *P. vulgaris* in Kenya after being evaluated for genetic stability, capacity to nodulate *P. vulgaris* in the presence of background rhizobia, and for properties critical to inoculant manufacture such as survival on carriers. A similar approach of inoculant development from native strains has been successfully employed in Brazil for *P. vulgaris* [24].

Isolates belonging to *R. phaseoli* and *R. paranaense* fixed more N_2_ in comparison to those in other groups. This observation was similar to findings in Brazil where effective *P. vulgaris* isolates belong to a limited number of species closely related to *R. tropici* [14], [24], [37], [44]. Taxa-related variation in N_2_ fixation in *Rhizobium* spp. could be due to differences in chromosomal or plasmid-borne symbiotic genes. We found that *nodC* alleles may be linked to N_2_ fixation outcomes with *nodC* type γ-b associated with a reduced capacity to fix N_2_ with *P. vulgaris* cv. KK08. However, it is unclear how the *nodC* polymorphisms may lead to differences in N_2_ fixation and further investigations are required.

## Conclusions

*P. vulgaris* has been cultivated in Kenya for ca. 500 years only [22] and our findings on the phylogeny of the isolates give valuable insights into the distribution, survival, and evolution of *P. vulgaris* microsymbionts in an area of recent host introduction. The taxa recovered in this study are not known to commonly nodulate *P. vulgaris* in other *P. vulgaris*-growing countries and these findings highlight the need for continued investigations into *P. vulgaris* microsymbionts in areas where the crop is of economic importance, as a wide array of interactions exist with this promiscuously-nodulating host. Better characterisation of native rhizobial populations in these areas will enable the development of management interventions that maximise inputs from symbiotic N_2_ fixation.
