# Supplementary material for: Genetic diversity and symbiotic effectiveness of Phaseolus vulgaris-nodulating rhizobia in Kenya
Source: Syst Appl Microbiol. 2018 Jul;41(4):291–9. doi: 10.1016/j.syapm.2018.02.001 (PMC6052332; doi:10.1016/j.syapm.2018.02.001)
Supplement: Supplementary file 1 [file mmc1.pdf]

Systematic and Applied Microbiology

Supplementary data for:

Genetic diversity and symbiotic effectiveness of *Phaseolus vulgaris*-nodulating rhizobia in Kenya

George M. Mwenda*, Graham W. O'Hara, Sofie E. De Meyer, John G. Howieson, Jason J. Terpolilli

Centre for Rhizobium Studies, Murdoch University, 90 South Street, Murdoch WA 6150, Australia

*Corresponding author, email: george.mwenda@murdoch.edu.au

Table S1: The geographical origin of isolates recovered from *P. vulgaris* in Kenya, their RP01-PCR groupings, effectiveness and GenBank accession numbers of sequenced genes. Accession numbers of reference sequences from GenBank are also included.

Isolate/Strain	Geographical origin in Kenya	Site	RPO1-PCR Group	RFLP 16S rRNA group	16S rRNA	*recA*	*atpD*	*nodC*	Effectiveness**
NAK 103	Butula, Busia	1	49	2	KY921857	MF065950	MG288740	MF065968	Effective
NAK 104	Butula, Busia	1	16	3	MF623859	MG288718	MG288741	MG288696	Effective
NAK 108	Butula, Busia	1	51	–	–	–	–	–	–
NAK 209	Loresho, Nairobi	2	71	–	–	–	–	–	–
NAK 210	Loresho, Nairobi	2	55	1	KY921860	MF065953	MG288743	MF065970	Ineffective
NAK 211	Loresho, Nairobi	2	55	–	–	–	–	–	–
NAK 212	Loresho, Nairobi	2	77	–	–	–	–	–	–
NAK 213	Loresho, Nairobi	2	71	–	–	–	–	–	–
NAK 214	Loresho, Nairobi	2	4	1	MF623860	MG288719	MG288744	MG288697	Effective
NAK 215	Loresho, Nairobi	2	1	–	–	–	–	–	–
NAK 216	Loresho, Nairobi	2	58	1	–	–	–	–	–
NAK 217	Loresho, Nairobi	2	58	–	–	–	–	–	–
NAK 218	Loresho, Nairobi	2	49	–	–	–	–	–	–
NAK 219	Loresho, Nairobi	2	74	–	–	–	–	–	–
NAK 220	Loresho, Nairobi	2	49	2	MF623861	MG288720	MG288745	MG288698	Effective
NAK 221	Loresho, Nairobi	2	53	–	–	–	–	–	–
NAK 222	Loresho, Nairobi	2	74	–	–	–	–	–	–
NAK 223	Kabete, Kiambu	3	26	2	–	–	–	–	–
NAK 224	Kabete, Kiambu	3	53	–	–	–	–	–	–
NAK 225	Kabete, Kiambu	3	49	–	–	–	–	–	–
NAK 226	Kabete, Kiambu	3	32	2	–	–	–	–	–
NAK 227	Kabete, Kiambu	3	56	1	MF623863	MG288721	MG288746	MG288699	Effective

Isolate/Strain	Geographical origin in Kenya	Site	RPO1-PCR Group	RFLP 16S rRNA group	16S rRNA	*recA*	*atpD*	*nodC*	Effectiveness**
NAK 228	Kabete, Kiambu	3	81	2	–	–	–	–	–
NAK 229	Kabete, Kiambu	3	57	–	–	–	–	–	–
NAK 230	Kabete, Kiambu	3	56	–	–	–	–	–	–
NAK 231	Kabete, Kiambu	3	30	2	MF623864	MG288722	MG288747	MG288700	Poor
NAK 232	Kabete, Kiambu	3	83	2	–	–	–	–	–
NAK 233	Kabete, Kiambu	3	56	1	–	–	–	–	–
NAK 234	Kabete, Kiambu	3	36	2	–	–	–	–	–
NAK 235	Kabete, Kiambu	3	57	–	–	–	–	–	–
NAK 237	Kabete, Kiambu	3	74	1	–	–	–	–	–
NAK 238	Kabete, Kiambu	3	56	–	–	–	–	–	–
NAK 239	Kabete, Kiambu	4	49	2	KY921861	MF065954	MG288748	MF065971	Effective
NAK 240	Kabete, Kiambu	4	49	–	–	–	–	–	–
NAK 241	Kabete, Kiambu	4	49	–	–	–	–	–	–
NAK 242	Kabete, Kiambu	4	49	2	MF623865	MG288723	MG288749	MG288701	Partial
NAK 243	Kabete, Kiambu	4	49	–	–	–	–	–	–
NAK 244	Kabete, Kiambu	4	49	–	–	–	–	–	–
NAK 245	Wangige, Kiambu	5	78	2	KY921862	MF065955	MG288750	MF065972	Partial
NAK 246	Wangige, Kiambu	5	59	–	–	–	–	–	–
NAK 247	Wangige, Kiambu	5	68	1	–	–	–	–	–
NAK 248	Wangige, Kiambu	5	46	2	–	–	–	–	–
NAK 249	Wangige, Kiambu	5	79	2	–	–	–	–	–
NAK 251	Ndenderu, Kiambu	6	5	–	–	–	–	–	–
NAK 252	Ndenderu, Kiambu	6	5	1	–	–	–	–	–
NAK 253	Ndenderu, Kiambu	6	84	1	–	–	–	–	–
NAK 254	Ndenderu, Kiambu	6	20	1	MF623866	MG288724	MG288751	MG288702	Effective

Isolate/Strain	Geographical origin in Kenya	Site	RPO1-PCR Group	RFLP 16S rRNA group	16S rRNA	*recA*	*atpD*	*nodC*	Effectiveness**
NAK 255	Ndenderu, Kiambu	6	59	1	–	–	–	–	–
NAK 256	Ndenderu, Kiambu	6	33	2	–	–	–	–	–
NAK 257	Ndenderu, Kiambu	6	62	1	–	–	–	–	–
NAK 258	Ndenderu, Kiambu	6	60	1	–	–	–	–	–
NAK 259	Ndenderu, Kiambu	6	65	1	–	–	–	–	–
NAK 260	Ndenderu, Kiambu	6	59	–	–	–	–	–	–
NAK 261	Ndenderu, Kiambu	6	58	–	–	–	–	–	–
NAK 262	Ndenderu, Kiambu	6	5	–	–	–	–	–	–
NAK 263	Ndenderu, Kiambu	6	45	–	–	–	–	–	–
NAK 264	Ndenderu, Kiambu	7	53	–	–	–	–	–	–
NAK 265	Ndenderu, Kiambu	7	37	–	–	–	–	–	–
NAK 266	Ndenderu, Kiambu	7	59	1	KY921863	MF065956	MG288752	MF065973	Partial
NAK 267	Ndenderu, Kiambu	7	53	–	–	–	–	–	–
NAK 268	Ndenderu, Kiambu	7	58	–	–	–	–	–	–
NAK 269	Ndenderu, Kiambu	7	37	–	–	–	–	–	–
NAK 270	Ndenderu, Kiambu	7	39	1	–	–	–	–	–
NAK 271	Ndenderu, Kiambu	7	37	–	–	–	–	–	–
NAK 272	Ndenderu, Kiambu	7	37	–	–	–	–	–	–
NAK 273	Ndenderu, Kiambu	7	37	–	–	–	–	–	–
NAK 274	Ndenderu, Kiambu	7	80	–	–	–	–	–	–
NAK 275	Ndenderu, Kiambu	7	80	2	–	–	–	–	–
NAK 276	Ndenderu, Kiambu	7	82	1	–	–	–	–	–
NAK 277	Ndenderu, Kiambu	7	7	1	–	–	–	–	–
NAK 278	Ndenderu, Kiambu	7	37	–	–	–	–	–	–
NAK 279	Ndenderu, Kiambu	7	38	1	–	–	–	–	–

Isolate/Strain	Geographical origin in Kenya	Site	RPO1-PCR Group	RFLP 16S rRNA group	16S rRNA	*recA*	*atpD*	*nodC*	Effectiveness**
NAK 280	Ndenderu, Kiambu	8	19	1	–	–	–	–	–
NAK 281	Ndenderu, Kiambu	8	63	1	–	–	–	–	–
NAK 282	Ndenderu, Kiambu	8	8	1	–	–	–	–	–
NAK 283	Ndenderu, Kiambu	8	8	–	–	–	–	–	–
NAK 284	Ndenderu, Kiambu	8	72	1	MF623868	MG288725	MG288753	MG288703	Effective
NAK 285	Ndenderu, Kiambu	8	72	–	–	–	–	–	–
NAK 286	Ndenderu, Kiambu	8	47	–	–	–	–	–	–
NAK 287	Ndenderu, Kiambu	8	31	2	KY921864	MF065957	MG288754	MF065974	Effective
NAK 288	Ndenderu, Kiambu	8	48	1	MF623869	MG288726	MG288755	MG288704	Effective
NAK 289	Ndenderu, Kiambu	8	59	–	–	–	–	–	–
NAK 290	Ndenderu, Kiambu	8	54	1	–	–	–	–	–
NAK 291	Ndenderu, Kiambu	8	50	–	–	–	–	–	–
NAK 292	Ndenderu, Kiambu	8	74	–	–	–	–	–	–
NAK 293	Ndenderu, Kiambu	8	59	–	–	–	–	–	–
NAK 294	Ndenderu, Kiambu	8	50	4	KY921865	MF065958	MG288756	MF065975	Effective
NAK 295	Muchatha, Kiambu	9	45	1	MF623870	MG288727	MG288757	MG288705	Effective
NAK 296	Muchatha, Kiambu	9	59	–	–	–	–	–	–
NAK 297	Muchatha, Kiambu	9	64	1	–	–	–	–	–
NAK 298	Muchatha, Kiambu	9	61	1	–	–	–	–	–
NAK 299	Muchatha, Kiambu	9	46	2	MF623871	MG288728	MG288758	MG288706	Effective
NAK 300	Muchatha, Kiambu	9	59	–	–	–	–	–	–
NAK 301	Muchatha, Kiambu	9	59	–	–	–	–	–	–
NAK 302	Muchatha, Kiambu	9	59	–	–	–	–	–	–
NAK 303	Muchatha, Kiambu	9	6	1	–	–	–	–	–
NAK 304	Muchatha, Kiambu	9	46	–	–	–	–	–	–

Isolate/Strain	Geographical origin in Kenya	Site	RPO1-PCR Group	RFLP 16S rRNA group	16S rRNA	*recA*	*atpD*	*nodC*	Effectiveness**
NAK 305	Muchatha, Kiambu	9	59	–	–	–	–	–	–
NAK 306	Muchatha, Kiambu	9	48	–	–	–	–	–	–
NAK 307	Muchatha, Kiambu	9	59	–	–	–	–	–	–
NAK 308	Muchatha, Kiambu	9	28	1	–	–	–	–	–
NAK 309	Muchatha, Kiambu	9	29	1	–	–	–	–	–
NAK 310	Limuru, Kiambu	10	38	–	–	–	–	–	–
NAK 311	Limuru, Kiambu	10	37	–	–	–	–	–	–
NAK 312	Limuru, Kiambu	10	1	1	KY921866	MF065959	MG288759	MF065976	Partial
NAK 313	Limuru, Kiambu	10	77	1	–	–	–	–	–
NAK 314	Limuru, Kiambu	10	2	–	–	–	–	–	–
NAK 315	Limuru, Kiambu	10	71	1	MF623873	MG288729	MG288760	MG288707	Partial
NAK 316	Limuru, Kiambu	10	12	1	–	–	–	–	–
NAK 317	Limuru, Kiambu	10	3	1	–	–	–	–	–
NAK 318	Limuru, Kiambu	10	63	–	–	–	–	–	–
NAK 319	Limuru, Kiambu	10	46	–	–	–	–	–	–
NAK 320	Limuru, Kiambu	10	12	–	–	–	–	–	–
NAK 321	Limuru, Kiambu	10	37	1	MF623874	MG288730	MG288761	MG288708	Partial
NAK 322	Limuru, Kiambu	10	63	–	–	–	–	–	–
NAK 324	Limuru, Kiambu	10	13	1	–	–	–	–	–
NAK 325	Limuru, Kiambu	10	73	1	–	–	–	–	–
NAK 326	Tigoni, Kiambu	11	42	1	–	–	–	–	–
NAK 327	Tigoni, Kiambu	11	24	1	MF623875	MG288731	MG288762	MG288709	Effective
NAK 328	Tigoni, Kiambu	11	42	–	–	–	–	–	–
NAK 329	Tigoni, Kiambu	11	43	1	–	–	–	–	–
NAK 330	Tigoni, Kiambu	11	25	1	–	–	–	–	–

Isolate/Strain	Geographical origin in Kenya	Site	RPO1-PCR Group	RFLP 16S rRNA group	16S rRNA	*recA*	*atpD*	*nodC*	Effectiveness**
NAK 331	Tigoni, Kiambu	11	23	–	–	–	–	–	–
NAK 332	Tigoni, Kiambu	11	21	1	MF623876	MG288732	MG288763	MG288710	Poor
NAK 333	Tigoni, Kiambu	11	21	–	–	–		–	
NAK 334	Tigoni, Kiambu	11	10	1	KY921867	MF065960	MG288764	MF065977	Partial
NAK 335	Tigoni, Kiambu	11	23	–	–	–	–	–	–
NAK 336	Tigoni, Kiambu	11	22	–	–	–	–	–	–
NAK 337	Tigoni, Kiambu	11	11	–	–	–	–	–	–
NAK 338	Tigoni, Kiambu	11	27	1	–	–	–	–	–
NAK 339	Tigoni, Kiambu	11	22	–	–	–	–	–	–
NAK 340	Tigoni, Kiambu	11	23	1	–	–	–	–	–
NAK 341	Tigoni, Kiambu	11	2	–	–	–	–	–	–
NAK 342	Limuru, Kiambu	12	17	–	–	–	–	–	–
NAK 343	Limuru, Kiambu	12	17	3	MF623877	MG288733	MG288765	MG288711	Effective
NAK 344	Limuru, Kiambu	12	17	–	–	–	–	–	–
NAK 345	Limuru, Kiambu	12	34	1	–	–	–	–	–
NAK 346	Limuru, Kiambu	12	6	–	–	–	–	–	–
NAK 347	Limuru, Kiambu	12	17	–	–	–	–	–	–
NAK 349	Limuru, Kiambu	12	17	3	KY921868	MF065961	MG288766	MF065978	Effective
NAK 350	Kiambu	13	76	2	–	–	–	–	–
NAK 351	Kiambu	13	9	2	–	–	–	–	–
NAK 352	Kiambu	13	81	–	–	–	–	–	–
NAK 354	Kiambu	13	35	3	MF623879	MG288734	MG288767	MG288712	Effective
NAK 355	Kiambu	13	80	–	–	–	–	–	–
NAK 356	Ruaka, Kiambu	14	37	–	–	–	–	–	–
NAK 357	Ruaka, Kiambu	14	37	–	–	–	–	–	–

Isolate/Strain	Geographical origin in Kenya	Site	RPO1-PCR Group	RFLP 16S rRNA group	16S rRNA	*recA*	*atpD*	*nodC*	Effectiveness**
NAK 358	Ruaka, Kiambu	14	2	1	KY921870	MF065963	MG288768	MF065979	Partial
NAK 359	Ruaka, Kiambu	14	15	1	–	–	–	–	–
NAK 360	Ruaka, Kiambu	14	44	2	–	–	–	–	–
NAK 361	Ruaka, Kiambu	14	38	–	–	–	–	–	–
NAK 362	Ruaka, Kiambu	14	40	–	–	–	–	–	–
NAK 363	Kinoru,Meru	15	14	1	MF623880	MG288735	MG288769	MG288713	Effective
NAK 364	Kinoru,Meru	15	71	–	–	–	–	–	–
NAK 365	Kinoru,Meru	15	66	–	–	–	–	–	–
NAK 366	Kinoru,Meru	15	67	–	–	–	–	–	–
NAK 367	Kinoru,Meru	15	41	1	–	–	–	–	–
NAK 368	Kinoru,Meru	15	67	2	MF623882	MG288736	MG288770	MG288714	Partial
NAK 369	Kinoru,Meru	15	52	1	–	–	–	–	–
NAK 370	Kinoru,Meru	15	62	–	–	–	–	–	–
NAK 372	Kinoru,Meru	15	66	–	–	–	–	–	–
NAK 373	Kinoru,Meru	15	69	–	–	–	–	–	–
NAK 374	Kinoru,Meru	15	66	–	–	–	–	–	–
NAK 375	Kinoru,Meru	15	70	1	–	–	–	–	–
NAK 376	Kinoru,Meru	15	66	–	–	–	–	–	–
NAK 377	Kinoru,Meru	15	66	–	–	–	–	–	–
NAK 378	Kinoru,Meru	15	66	2	MF623883	MG288737	MG288771	MG288715	Effective
NAK 379	Kinoru,Meru	15	18	–	–	–	–	–	–
NAK 380	Kinoru,Meru	15	71	–	–	–	–	–	–
NAK 381	Kinoru,Meru	15	71	–	–	–	–	–	–
NAK 382	Kinoru,Meru	15	75	1	KY921871	MF065964	MG288772	MF065980	Partial
NAK 383	Kinoru,Meru	15	75	–	–	–	–	–	–

Isolate/Strain	Geographical origin in Kenya	Site	RPO1-PCR Group	RFLP 16S rRNA group	16S rRNA	*recA*	*atpD*	*nodC*	Effectiveness**
NAK 385	Kinoru,Meru	15	71	1	–	–	–	–	–
NAK 386	Kinoru,Meru	15	48	–	–	–	–	–	–
NAK 387	Kinoru,Meru	15	66	2	KY921873	MF065966	MG288773	MF065981	Partial
NAK 407	Sidada, Siaya	16	85	2	MF623886	MG288738	MG288774	MG288716	Effective
NAK 440	Sidada, Siaya	16	86	2	MF623887	MG288739	MG288775	MG288717	Poor
NAK 458	Sidada, Siaya	16	87	2	KY921874	MF065967	MG288776	MF065982	Effective
B. japonicum USDA 6^T					AP01220	AM168341.1	AM168320.1	AP012206	
R. acidisoli FH13^T					KJ921033	KJ921098.1	KJ921069.1	KJ921061	
R. aegyptiacum 1010^T					JQ670243.3	KU664569.1	KU664561.1	KU664566.1	
R. alamii GBV016^T					AM931436.1	–	–	–	
R. altiplani BR 10423^T					KX022634.1	–	–	–	
R. anhuiense CCBAU 23252^T					KF111868	JQ585891.1	JQ585863.1	–	
R. bangladeshense BLR175^T					JN648931.2	JN649057.1	JN648967.1	–	
R. binae BLR195^T					JN648932.2	JN649058.1	JN648968.1	–	
R. calliandrae CCGE 524^T					JX855162	JX855189.1	JX855199.1	–	

Isolate/Strain	Geographical origin in Kenya	Site	RPO1-PCR Group	RFLP 16S rRNA group	16S rRNA	*recA*	*atpD*	*nodC*	Effectiveness**
R. ecuadorense CNPSO 671^T					LFIO01000095	JN129351.1	LFIO01001065.1	LFIO01000036.1	
R. etli CFN 42^T/ USDA 9032^T					CP000133	CP000133.1	AJ294404.1	U80928	
R. etli CIAT 652					–	–	–	CP001076	
R. etli KIM5					–	–	–	CP021125.1	
R. fabae CCBAU 33202^T					DQ835306	EF579941.1	EF579929.1	JN580683	
R. favelukesii LPU83^T					HG916852	–	–	–	
R. freirei PRF 81^T					AQHN01000056	AQHN01000010.1	AQHN01000005	AQHN01000086.1	
R. gallicum R602sp^T					ARDC01000036	AY907357.1	HM142762.1	AF217266	
R. grahamii CCGE 502^T					AEYE01000061	JF424622.1	JF424612.1	JN021932.1	
R. hainanense CCBAU 57015^T/I66^T					FMAC01000030	HQ394252.1	HQ394217.1	–	
R. indigoferae CCBAU 71042^T					NR025157.1	–	–	–	
R. jaguaris CCGE 525^T					JX855169	JX855192.1	JX855202.1	–	
R. laguerreae FB206^T					JN558651	JN558681.2	JN558661.1	KC608575	

Isolate/Strain	Geographical origin in Kenya	Site	RPO1-PCR Group	RFLP 16S rRNA group	16S rRNA	*recA*	*atpD*	*nodC*	Effectiveness**
R. leguminosarum USDA 2370^{T}					U29386	AJ294376	AJ294405.1	FJ596038	
R. lentis BLR27^{T}					JN648905.2	JN649031.1	JN648941.1	–	
R. leucaenae USDA 9039^{T}					AUFB01000074	AJ294372	AJ294396.1	AUFB01000059.1	
R. loessense CCBAU 7190B^{T}					NR115125.1	–	–	–	
R. lusitanum P1-7^{T}					AY738130	DQ431674	DQ431671.1	HM852098	
R. mayense CCGE 526^{T}					JX855172	JX855195.1	JX855205.1	–	
R. mesoamericanum CCGE 501^{T}					–	–	–	JN021931.1	
R. mesosinicum CCBAU 25010^{T}					NR043548.1	–	–	–	
R. miluonense HAMBI 2971^{T}					jgi.1052910	HM047131.1	HM047116.1	–	
R. mongolense USDA 1844^{T}					ATTQ01000080	–	–	–	
R. multihospitium HAMBI 2975^{T}/ CCBAU 83401^{T}					jgi.1052913	EF490029	EF490019.1	EF050781	

Isolate/Strain	Geographical origin in Kenya	Site	RPO1-PCR Group	RFLP 16S rRNA group	16S rRNA	*recA*	*atpD*	*nodC*	Effectiveness**
R. paranaense PRF 35^T					EU488753	EU488826.1	–	–	
R. phaseoli ATCC 14482^T					EF141340	EF113136	EF113151.1	HM441255	
R. pisi DSM 30132^T					AY509899	DQ431676	EF113149.1	JQ795195	
R. rhizogenes NBRC 13257^T					BAYX01000035	AJ294374.1	AJ294398.1	–	
R. sophorae CCBAU 03386^T					KJ831229	KJ831252	KJ831235.1	KJ831243	
R. sophoriradicis CCBAU 03470^T					KJ831225	KJ831248	KJ831231.1	KJ831245	
R. sullae pIS 123^T					Y10170.1	FJ816279.1	DQ345069.1	–	
R. tibeticum CCBAU 85039^T					NR116254.1	–	–	–	
R. trifolii ATCC 14480^T					–	–	–	FJ895269	
R. tropici CIAT 899^T/ USDA 9030^T					CPOO4015	CP004015	AJ294397.1	JN580681	
R. vallis CCBAU 65647^T					FJ839677	GU211770	GU211768.1	GU211769	
R. yanglingense SH22623^T					NR028663.1	–	–	–	

Isolate/Strain	Geographical origin in Kenya	Site	RPO1-PCR Group	RFLP 16S rRNA group	16S rRNA	*recA*	*atpD*	*nodC*	Effectiveness**

** Effective (≥75% of CIAT 899 SDW), partially effective (74%-50%), poorly effective (49-25%), or ineffective (≤24%)

Table S2: Oligonucleotide primers, PCR reaction mixes and PCR cycling conditions used in this study

Gene	Primer sequence (5′-3′)	Ref	PCR reaction mix	Cycling conditions
DNA fingerprinting	RP01: AATTTTCAAGCGTCGTGCCA	(4)	20 µL PCR: 1× PCR polymerization buffer (Fisher Biotec), 3 mM $MgCl_2$, 1.25 µM RP01 primer, 2.5 U Taq DNA polymerase, 1 µL DNA and PCR water	5× (94°C for 30 s, 50°C for 10 s, 72°C for 90 s); 35× (94°C for 30 s, 55°C for 25 s, 72°C for 90 s); 72°C for 5 min
16S rRNA	27F: AGAGTTTGATCCTGGCTCAG<br>1492R: ACGGCTACCTTGTTACGACTT	(3)	25 µL PCR: 1× GoTaq® Green Master Mix, 0.4 µM 27F primer, 0.4 µM 1492R primer, 2 µL DNA and PCR water	94°C for 120 s; 35× (94°C for 30 s, 55°C for 25 s, 72°C for 90 s); 72°C for 7 min
recA	6F: CGKCTSGTAGAGGAYAAATCGGTGGA<br>555R: CGRATCTGGTTGATGAAGATCACCAT	(2)	25 µL PCR: 1× GoTaq® Green Master Mix, 0.4 µM 6F primer, 0.4 µM 555R primer, 2 µL DNA and PCR water	94°C for 120 s, 32× (94°C for 45 s, 60°C for 60 s, 72°C for 60 s); 72°C for 5 min
atpD	273F: SCTGGGSCGYATCMTGAACGT<br>771R: GCCGACACTTCCGAACCNGCCTG	(2)	25 µL PCR: 1× GoTaq® Green Master Mix, 0.4 µM 273F primer, 0.4 µM 771R primer, 2 µL DNA and PCR water	94°C for 120 s, 32× (94°C for 45 s, 60°C for 60 s, 72°C for 60 s); 72°C for 5 min
nodC	540F: TGATYGAYATGGARTAYTGGC<br>1164R: GAYARCCARTCGCTRTTG	(1)	25 µL PCR: 1× GoTaq® Green Master Mix, 0.4 µM 540F primer, 0.4 µM 1164R primer, 2 µL DNA and PCR water	94°C for 120 s; 3× (94°C for 60 s, 50°C for 135 s, 72°C for 60 s); 30× (94°C for 35 s, 50°C for 75 s, 72°C for 75 s); 72°C for 7 min

NB: K = G or T; S = G or C; R = A or G; Y = C or T; M = A or C; N = A, T, G, or C

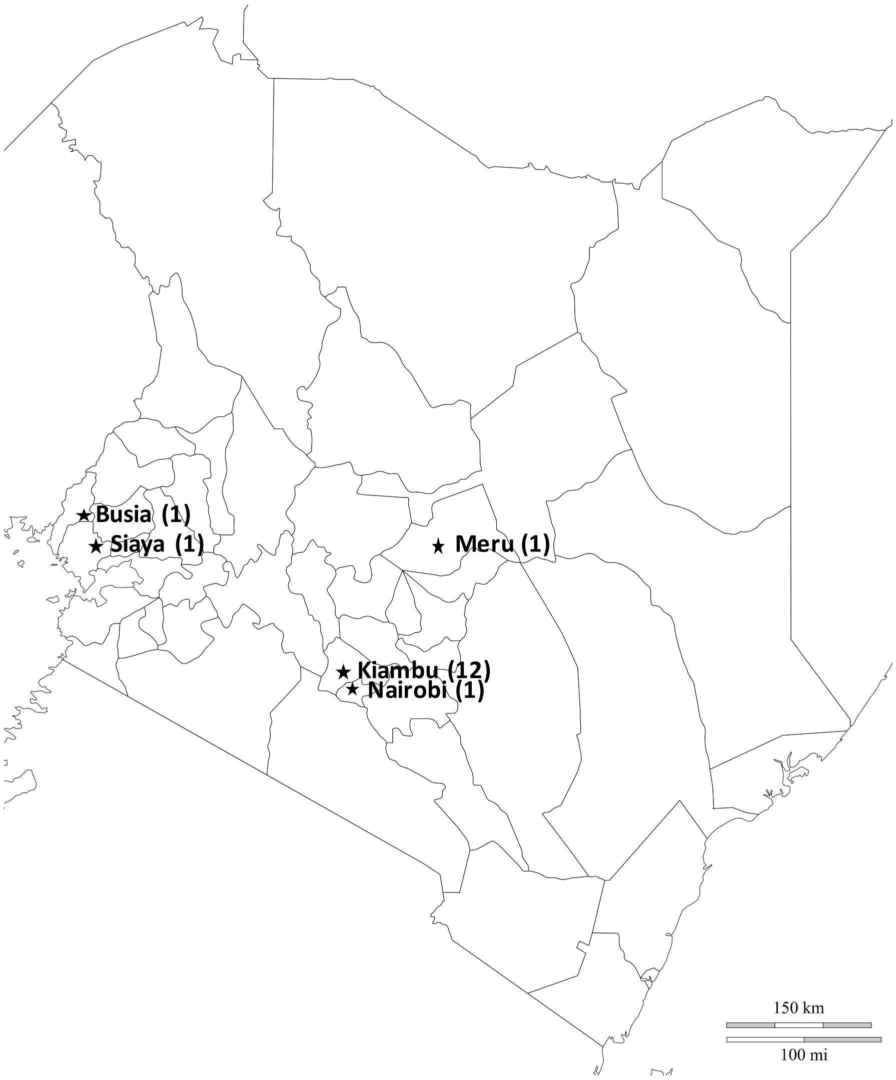

Figure S1: A map of Kenya showing the origin of rhizobia used in this study (number of sampling sites in each county is given in parenthesis). The generalized altitude, annual rainfall and soil types in the sampling areas are: **Busia** and **Siaya-** 1200-1500 m.a.s.l, 1200-2000 mm, acrisols and ferralsols; **Kiambu** and **Nairob**i- 1600-2000 m.a.s.l, 1200-1800 mm, humic nitisols; and **Meru-** 1700 m.a.s.l, 1500-2000 mm, cambisols.

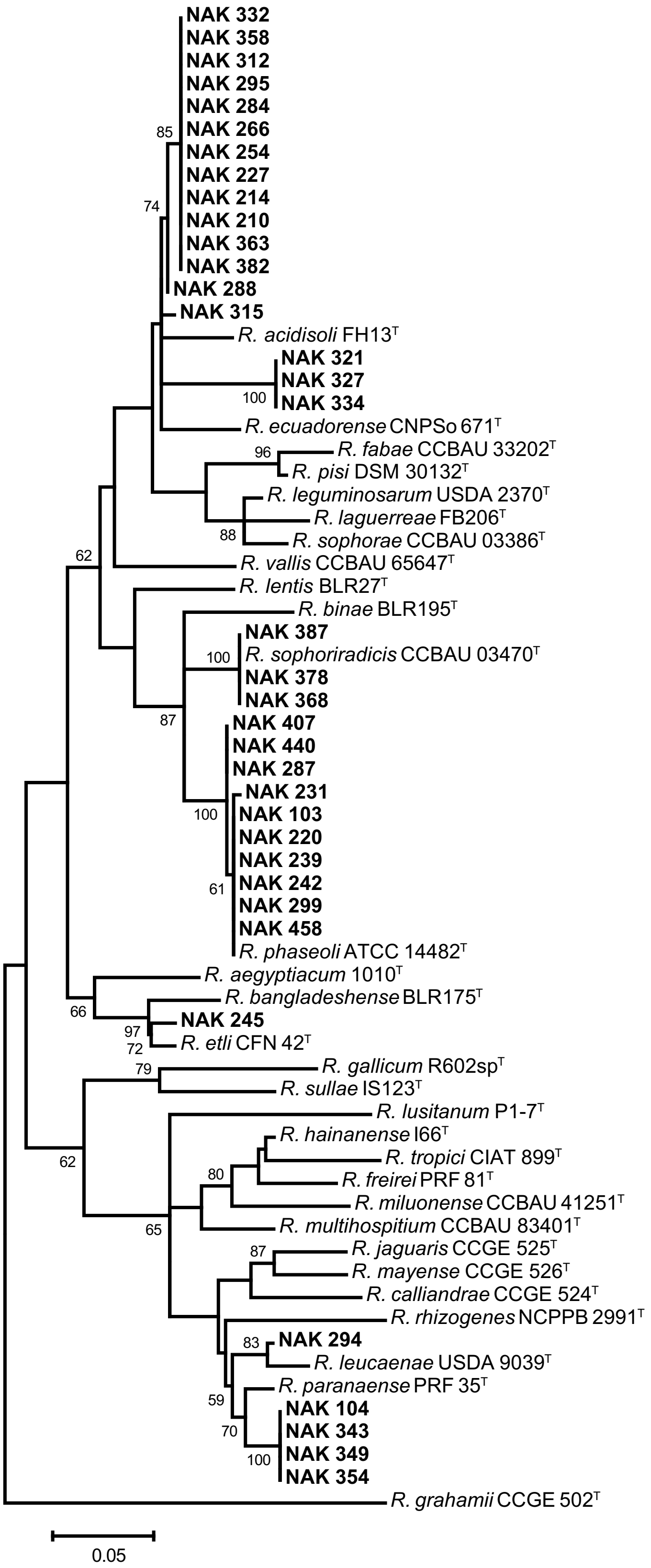

Figure S2: Maximum Likelihood phylogeny of the *recA* gene showing the relationship between 36 rhizobial isolates from *P. vulgaris* cultivated in Kenya (in bold) and their closest type strains. There was a total of 340 positions in the final dataset. Node supports higher than 50% are labelled with a bootstrap value (1000 replicates). Bar indicates five nucleotide substitutions per 100 nucleotides.

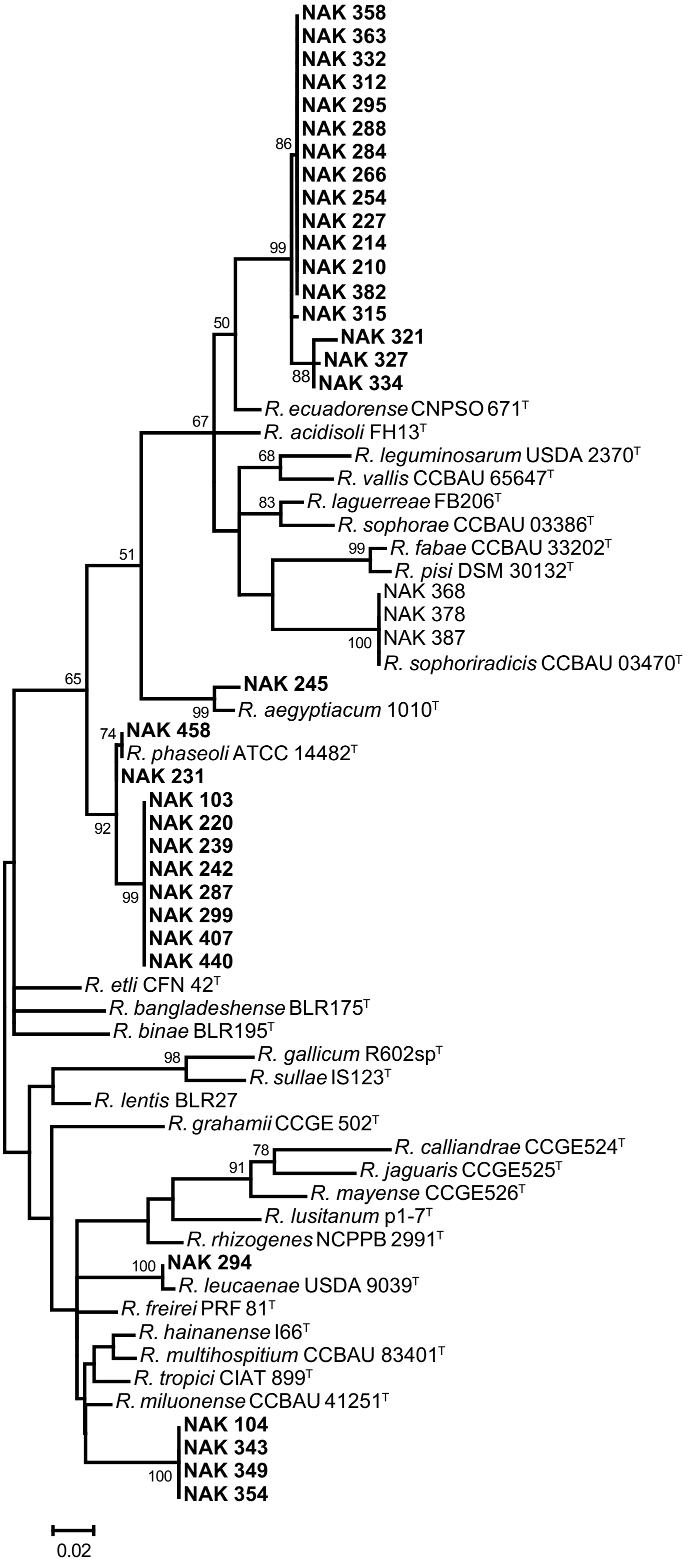

Figure S3: Maximum Likelihood phylogeny of the *atpD* gene showing the relationship between 36 rhizobial isolates from *P. vulgaris* cultivated in Kenya (in bold) and their closest type strains. There was a total of 396 positions in the final dataset. Node supports higher than 50% are labelled with a bootstrap value (1000 replicates). Bar indicates two nucleotide substitutions per 100 nucleotides.

Supplementary references:

1. De Meyer SE, Van Hoorde K, Vekeman B, Braeckman T, Willems A. 2011. Genetic diversity of rhizobia associated with indigenous legumes in different regions of Flanders (Belgium). Soil Biology and Biochemistry 43:2384-2396.
2. Gaunt MW, Turner SL, Rigottier-Gois L, Lloyd-Macgilp SA, Young JPW. 2001. Phylogenies of *atpD* and *recA* support the small subunit rRNA-based classification of rhizobia. International Journal of Systematic and Evolutionary Microbiology 51:2037-2048.
3. Lane DJ. 1991. 16S/23S rRNA sequencing, p 115-147. *In* Stackebrandt E, Goodfellow M (ed), Nucleic Acids Techniques in Bacterial Systematics. John Wiley & Sons, Chichester.
4. Richardson AE, Viccars LA, Watson JM, Gibson AH. 1995. Differentiation of *Rhizobium* strains using the polymerase chain reaction with random and directed primers. Soil Biology and Biochemistry 27:515-524.
